# Preparation of Prussian Blue Containing Polymeric Nanocapsule via Interfacial Confined Coordination in Crosslinked Inverse Miniemulsion

**DOI:** 10.3390/polym11020266

**Published:** 2019-02-05

**Authors:** Lin Wu, Tao Pang, Yebin Guan, Yiguo Li

**Affiliations:** 1 Anhui Key Laboratory of Functional Coordination Compounds, School of Chemistry and Chemical Engineering, Anqing Normal University, Anqing, Anhui 246011, China; leileitaotao6666@163.com (T.P.); guanyb@aqnu.edu.cn (Y.G.); 2 Ningbo Key Laboratory of Specialty Polymers, Faculty of Materials Science and Chemical Engineering, Ningbo University, Ningbo 315211, China; 3 Anhui Collaborative Innovation Centre for Petrochemical New Materials, School of Chemistry and Chemical Engineering, Anqing Normal University, Anqing, Anhui 246011, China

**Keywords:** polymeric hybrid nanocapsule, Prussian blue, inverse miniemulsion, coordination

## Abstract

This work presents a simple and facile strategy for the creation of Prussian blue containing polymeric nanocapsules. An crosslinked inverse miniemulsion with a formula of water/ K_4_Fe(CN)_6_/1,2-bis-(-2-iodoethyl) ethane(BIEE)/ toluene/ PDMAEMA-*b*-PS stabilizer mixture was prepared as soft template firstly. A crosslinking nanocapsule structure with K_4_Fe(CN)_6_ in water core could be achieved by a crosslinking reaction between PDMAEMA-*b*-PS stabilizers and BIEE. Upon the following addition of FeCl_3_ ether solution into the oil phase of this inverse miniemulsion, a coordination reaction between two iron salts occurred immediately to form a Prussian blue complex. Due to the solubility limitation of FeCl_3_ in the oil phase of the miniemulsion, forcing the coordination reaction of K_4_Fe(CN)_6_ and FeCl_3_ mainly occurred at the oil-water interface of the nanocapsules, resulting in a soft polymer/Prussian blue(PB) hybrid nanocapsule.

## 1. Introduction

Polymeric inorganic hybrid materials have attracted much attention from scientific and technical applications due to their combination of the performance advantages of inorganic and polymeric materials. Hybrid materials exhibit superior stability, excellent biocompatibility and flexibility with a broad range of applications [1,2]. Particularly, they have been widely used in biotechnology applications, delivery, catalyst etc. [3,4,5,6,7,8]. 

To date, numbers of technique having been developed to synthesize polymer/inorganic hybrid nanostructures including self-assembly techniques [9,10,11], layer-by-layer deposition approach [12], (co)polymerization [13], etc. Wu and co-works synthesized hybrid macrocapsules using metal-organic coordination-enabled layer-by-layer self-assembly technique to prepare polymer inorganic hybrid materials [12]. Liu et al. reported the preparation of monodispersed soluble Prussian blue (PB) metal coordination nanospheres via confined metal coordination polymerization within the cores of P4VP-*b*-PDMA block copolymer [11].

Polymer/Prussian blue (PB) or PB analogue nanomaterials are some of the most widely studied polymeric inorganic hybrid materials, because of their various unusual properties, e.g. magnetism, conductivity [14,15]. Current efforts have led to a number of techniques for the creation of polymer/PB nanomaterials of various morphologies including sphere-like [16,17], cubic-like [18] and others [19]. Kitagawa and co-workers prepared soluble PB nanoparticles from a FeCl_2_ and K_3_(Fe(CN)_6_) aqueous solution mixture in the presence of polyvinylpyrrolidone [20]. Polymer/PB hollow spherical nanoshells were achieved by using miniemulsion periphery polymerization (MEPP) [18,21,22]. Surfactants with polymerizable end groups were used as stabilizers for the miniemulsion preparation and the subsequent nanoshell preparation. For example, Fe^3+^ ions underwent metal coordination at the periphery of the miniemulsion prepared using pentacyanoferate-terminated PEO-PPO-PEO triblock copolymer stabilizers, leading to the formation of Prussian blue nanoshell [21]. Recently, Liang et al. developed an approach for scalable synthesis of polymer/PB hybrid nanoparticles with tiny size through the direct disassembly-assisted synthesis strategy [23].

However, one of the drawbacks of these methods is that the preparation process of metallo-surfactant block copolymer is usually very tedious. To overcome this problem, we demonstrate a novel and simple two-step process for preparation PB containing hybrid polymeric nanocapsule by using crosslinked inverse miniemulsion as a soft template. In our previous study, we have developed a technique of miniemulsion crosslinking for the preparation of polymeric nanocapsule [24,25]. We found that PDMAEMA homopolymer or PDMAEMA-*b*-PB block copolymer as stabilizers in a miniemulsion could be easily crosslinked by BIEE crosslink under mild conditions to form a soft polymeric nanocapsule in one-pot reaction. Using this approach, in this case, the miniemulsion was replaced by an inverse miniemulsion, although block copolymer with PDMAEMA segments was also adopted and to be crosslinked by BIEE to form a crosslinked inverse miniemulsion. Herein, K_4_Fe(CN)_6_ as a co-stabilizer also plays a role as a reactant of PB and was wrapped into water droplets in an inverse miniemulsion. With the addition of the amount of FeCl_3_ diethyl ether solution into the oil phase of inverse miniemulsion, an in situ coordination reaction occurred at the surface of crosslinked inverse miniemulsion to generate a PB containing polymeric inorganic hybrid nanoparticle.

## 2. Materials and Methods

### 2.1. Materials

2-(Dimethyl amino)ethyl methacrylate(DMAEMA)(98%) was purchased from Sigma-Aldrich (St. Louis, MO, USA). Styrene (99%) was purchased from Aladdin Biochemical Technology Co., Ltd., (Shanghai, China). DMAEMA and styrene were passed through alumina column to remove inhibitor before use. All other reagents were used without further purification. Iron chloride(≥99.9%), potassium ferrocyanide trihydrate(>99%) and 2-cyano-2-propyl benzodithioate(>97%) were purchased from Aladdin Biochemical Technology Co., Ltd., (Shanghai, China). 1,2-Bis(2-iodoethoxy)ethane(BIEE) (>96%) was purchased from TCI Tokyo Chemical Industry Co., Ltd. (Tokyo, Japan). *n*-Hexane(≥97.0%) and toluene(≥99.5%) were purchased from Sinopharm Chemical Reagent Co., Ltd. (Shanghai, China). 

### 2.2. Characterization

^1^H-NMR (400 MHZ) spectra were recorded using a Bruker 400 UltraShield spectrometer (Bruker Biospin AG, Faellanden, Switzerland) at ambient temperature (about 20 °C) in CDCl_3_. The number average molecular weight (*M*_n_) and polydispersity index (PDI) was determined using size exclusion chromatography (SEC) equipped with a LC1120 HPLC pump (polymer laboratories, Shropshire, UK), a differential refractive index (DRI) detector (Shodex, RI-101, Showa Denko K.K., Kanagawa, Japan), a 5.0 μm bead-sized guard column (50 mm × 7.5 mm), and two PLgel 5.0 μm MIXED-C columns (300 mm × 7.5 mm) in series (Polymer laboratories, Shropshire, UK). Tetrahydrofuran (THF) with trimethylamine (TEA) (5%) and 2,6-di-tert-butyl-4-methylphenol (0.2 g) was used as the eluent at a flow rate of 1 mL·min^−1^ at ambient temperature. The SEC system was calibrated with polystyrene (ranging from 580 to 7,500,000 g·mol^−1^). The thermogravimetric analysis (TGA) curves were recorded using a TGA instrument (TGA-2900 model, TA instrument, Newcastle, DE, USA) thermo-balanced in a temperature range of 25–550 °C at a heating rate of 10 K/min under nitrogen. A transmission electron microscope (TEM; Philips CM-10, FEI Company, Eindhoven, The Netherlands) with an acceleration voltage of 80 kV was used to take low magnification TEM images and (JEOL—2010, Tokyo, Japan) with an acceleration voltage of 200 kV was used to take high magnification image and EDX mapping. TEM samples were prepared by dropping miniemulsion of methanol dilution onto a carbon-containing copper grid under ambient conditions. The specimens were observed with negative staining by using phosphotungstic acid (2%). A tapping mode atomic force microscope (AFM) (Keysight 5500 AFM/SPM System, Keysight Technologies, Inc., Santa Rosa, CA, USA) was employed to examine the height profiles on Mica slice. The AFM sample was prepared by dropping miniemulsion of methanol dilution on a 1 cm × 1 cm mica slice. The apparent hydrodynamic size of nanoparticles was measured using a Zetasizer (nano ZS90, Malvern Instruments, Worcestershire, UK) at 25 °C. The infrared spectra were recorded from 400 to 4000 cm^−1^ on an iS50FT-IR spectrometer (Thermo Scientific, Darmstadt, Germany) by using KBr pellets. UV–Vis spectra were recorded on an UV-2501PC spectrometer (ShimadzuCo., Kyoto, Japan).

### 2.3. Synthesis of Poly(N,N-(dimethylamino)ethyl methacrylate) Chain Transfer Agent (PDMAEMA-CTA)

The PDMAEMA-CTA with a targeted polymerization degree of 160 were prepared as follows: DMAEMA (11.32 g, 0.072 mol), 2-cyano-2-propyl benzodithioate (CPDB) (0.10 g, 0.45 mmol) and azobisisobutyronitrile (AIBN) (0.01g, 0.06 mmol), were dissolved in 2 mL of toluene. The mixture was purged with nitrogen for 30 min and then heated at 60 °C for 24 h. The reaction was quenched by rapid cooling with ice-water. After the removal of toluene using a rotary evaporator, the mixture was dissolved in THF and precipitated in *n*-hexane. The polymer was filtered and dried under vacuum at room temperature overnight yield final product of 9.17 g (81 %). The molecular weight (*M*_n_ = 25,000 g·mol^−1^) and molecular weight distribution (PDI = 1.20) of the product were determined by SEC with PS standards. ^1^H NMR (400 MHz, CDCl_3_, δ): 0.86-1.10 (d, 370 H, CCH_3_ from PDMAEMA), 1.81 (270 H, CCH_3_CH_2_ from PDMAEMA), 2.22 (s, 820 H, N(CH_3_)_2_ from PDMAEMA), 2.50(t, 266 H, NCH_2_CH_2_O from PDMAEMA), 3.99(t, 266 H, NCH_2_CH_2_O from PDMAEMA), 7.79(m, 2H, *o*-ArH, from chain transfer agent), 7.46(m, 1H, *p*-ArH, from chain transfer agent). Polymerization degree of PDMAEMA was determined to be 133. Number-average molecular weight of PDMAEMA-CTA was determined to be 157 g·mol^−1^ × 133 + 221 g·mol^−1^ = 21,102 g·mol^−1^.

### 2.4. Synthesis of PDMAEMA-b-PS

In a typical polymerization process, azobisisobutyronitrile (AIBN) (0.0035 g, 0.021 mmol) and PDMAEMA-CTA (4 g, 0.190 mmol) were dissolved in 5 mL of toluene, styrene (1.26 g, 0.012 mol) was then added into the flask. Subsequently, the above mixture was deoxygenated by purging with nitrogen gas for at least half an hour. The mixture was polymerized at 80 °C for 24 h. The polymerization was terminated by cooling in an ice bath. The product was then precipitated into methanol and the solid was collected by suction filtration (monomer conversion 40%). The molecular weight (*M*_n_ = 27,200 g·mol^−1^) and molecular weight distribution (PDI = 1.24) of the product were determined by SEC with PS standards. ^1^H NMR (400 MHz, CDCl_3_, δ): 0.86-1.10 (d, 378 H, CCH_3_ from PDMAEMA), 1.45 (br, 60H, CH_2_CH from PS), 1.84 (m, 225H, CH_2_CH, from PS and CCH_3_CH_2_ from PDMAEMA), 2.25 (s, 756H, N(CH_3_)_2_ from PDMAEMA), 2.54(t, 277H, NCH_2_CH_2_O from PDMAEMA), 4.03 (t, 257 H, NCH_2_CH_2_O from PDMAEMA), 6.25-7.20 (br, 58H, Ar H from PS).The ^1^H NMR spectrum agreed with literature data [26,27]. Polymerization degree of PS was determined to be 28. The number-average molecular weight of PDMAEMA-*b*-PS was determined to be 157 g·mol^−1^ × 133 + 104 g·mol^−1^ × 28 + 221 g·mol^−1^ = 24,014 g·mol^−1^. FTIR(KBr): υ(cm^−1^) 2940 (CH_3_, stretching), 2080(CH_2_, stretching of phenyl ring),1735 (C=O stretching), 1150 (C–N stretching).

### 2.5. Preparation of Crosslinked Inverse Miniemulsion

A representative preparation process is as follow; into a clear glass threaded vials (20 mL), PS-*b*-PDMAEMA (0.065 g, 0.0024 mmol) was dissolved in 6.5 g of toluene to produce a homogenous mixture. This organic phase was then added to the aqueous phase consisting of distilled water (0.65 g) and K_4_Fe(CN)_6_·3H_2_O (0.013 g, 0.031mmol, 2.0 wt % to water) under vigorous stirring. After 45 min, the resulting mixture was ultrasonicated at the power of 120 W (XO-SM50, Nanjing, China) for a period of 10 min while immersed in an ice bath to yield an inverse miniemulsion. BIEE (0.1 g, 0.27 mmol) crosslinker was introduced into inverse miniemulsion and the crosslinking reaction was stirred for 4 days at room temperature.

### 2.6. Preparation of PB Containing Polymeric Nanocapsule

The FeCl_3_ stock solution was prepared firstly by adding 0.40 g (2.5 mmol) of FeCl_3_ into 100 mL of diethyl ether. Varied amount of FeCl_3_ stock solution (0.0247 mol·L^−1^) was injected into the above miniemulsion according to the experimental design, and the reaction was stirred for 12 hours at room temperature before measurement.

## 3. Results and Discussions

### 3.1. Preparation and Analysis of Polymeric Nanocapsules

2-Cyano-2-propyl benzodithioate (CPDB) was used as a reversible addition fragmentation chain transfer agent for the controlled polymerization of *N,N*-(dimethylamino)ethyl methacrylate (DMAEMA). The resulting well-defined PDMAEMA-CTA was subsequently used as a macro-chain transfer agent for further polymerization of styrene, leading to the formation of block copolymer PDMAEMA-*b*-PS (Scheme S1, Supporting Information). The molecular weight, polydispersity and repeat units of PDMAEMA-CTA and PDMAEMA-*b*-PS were characterized by SEC and ^1^H NMR (Figure S1, Supporting Information), respectively.

The molecular weights, molecular weight distributions and monomer conversion of the resultant polymers are summarized in Table 1. The chain extension is evidenced by the molecular weight increase from homopolymers to block copolymers. In addition, both the homopolymer and copolymer have narrow PDIs (<1.24). Comparison of SEC with NMR methods for the measurement of the molecular weight of polymers, showed that the SEC analysis provided under estimated molecular weight values. This is because PS standards were used for the PDMAEMA homopolymer and block copolymer molecular weight calculation. Such use might not be truly appropriate. The resulted copolymer is an amphiphilic block copolymer, the PDMAEMA block is water soluble and PS block is oil soluble. As a result, PDMAEMA-*b*-PS block copolymers can be used as stabilizers in inverse miniemulsion preparation for the synthesis of nanocapsules *via* cross-linking reaction.

**Scheme 1:**
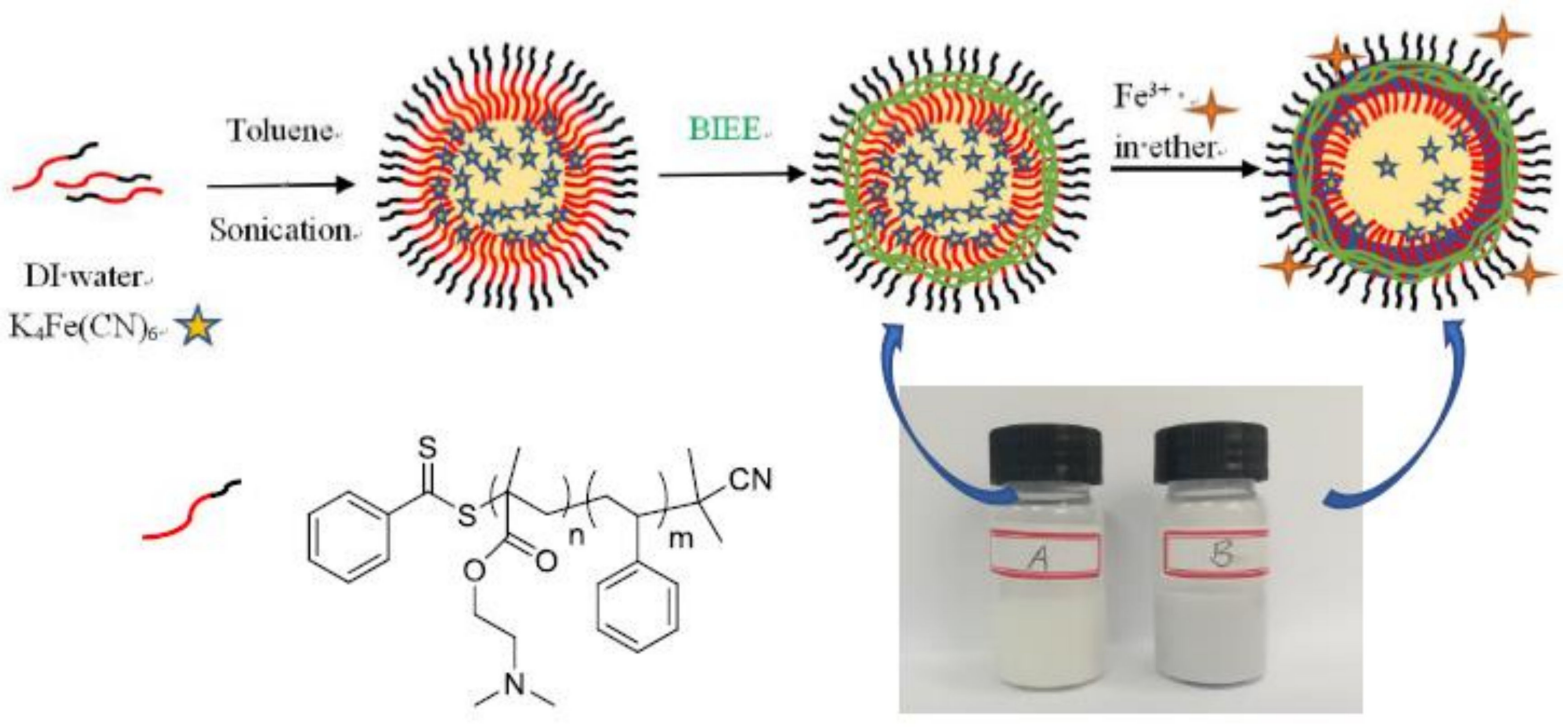
Schematic illustration for the preparation of PB containing polymeric nanocapsules. The inset shows digital photos of crosslinked miniemulsion before (left, A) and after (right, B) addition of Fe^3+^.

As shown in Scheme 1, the inverse miniemulsion were prepared from a mixture of toluene, PDMAEMA-*b*-PS, water and potassium ferrocyanide with ratio 90.1: 0.9: 8.8: 0.18 by weight. In them, PDMAEMA-*b*-PS was used as stabilizers in inverse miniemulsion. The tertiary amine groups of PDMAEMA block could further react with 1,2-bis(2-iodoethoxy)ethane(BIEE) crosslinker *via* quaternization reaction to form the polymeric shell of the nanocapsule. The crosslinking reaction was kept for 4 days at room temperature allowing the quaternization reaction to fix the nanocapsule structure. Here, K_4_Fe(CN)_6_ located in the water core acting as co-stabilizer in the inverse miniemulsion. After 4 days reaction, 0.1 g of above miniemulsion was diluted in 10 g of methanol, resulting a turbid liquid. The nanocapsule structure are expected to retain, although methanol is a good solvent for miscible with water and oil phase [21,24].

**Figure 1:**
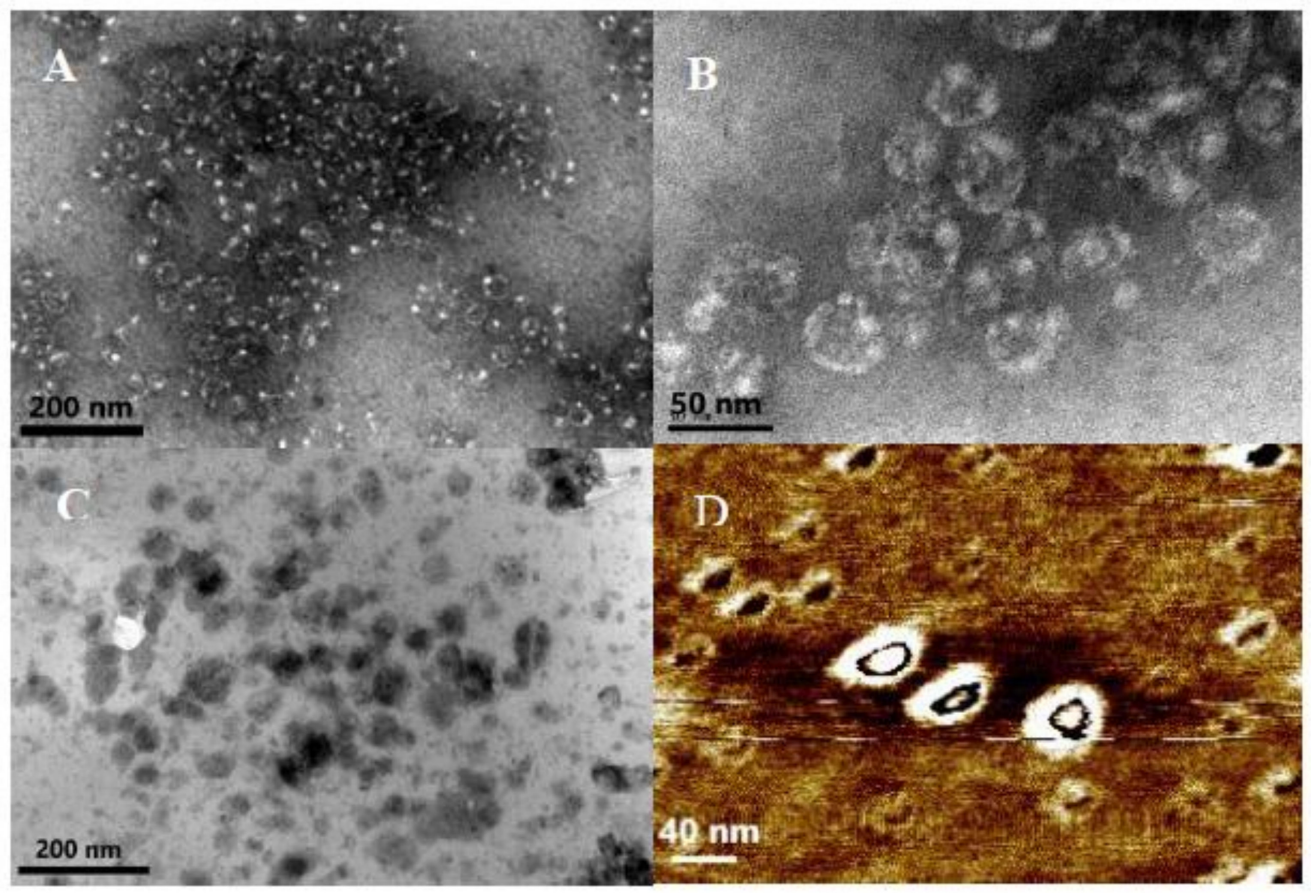
TEM images of nanocapsules prepared using PDMAEMA-b-PS as stabilizers with various phase fraction ((**A**,**B**): contained water phase 13 wt % to total weight; (**C**): contained water phase 23 wt % to total weight) dispersed in methanol and stained by phosphotungstic acid (2%) and height image of tapping mode AFM ((**D**): contained water phase 13 wt % to total weight).

The above turbid liquid was then characterized using TEM and AFM. The samples shown in Figure 1A–C were treated by phosphotungstic acid (2%) for negative staining. The samples of Figure 1A,B,D were prepared using PDMAEMA-*b*-PS as stabilizer with 13 wt % of water phases to total weight of emulsion. Here, spherical particles with diameter of approximately 40 nm was observed in Figure 1A. We also notice that the size distribution of particles is narrow. However, DLS measurement of the particles in a diluted methanol solution revealed a diameter of 188 nm with a polydispersity of 0.386 (Figure S2, Supporting Information). This conflicting result between DLS and TEM suggests a number of larger aggregates were formed in solution. It is caused by a poor solubility of PS segment of stabilizer of capsules in methanol making the capsules to be accumulated and DLS normally emphasizes larger particles. In Figure 1B, the particles exhibit a collapsed structure with a ring-like morphology which is a typical of hollow shell structure [21]. The AFM images (see Figure 1D and Figure S3A in Supporting Information) further verified the spherical nanocapsules. The dark color in the central part of the particles also indicate that the particles are center collapsed. Moreover, the thickness of the ring is ca. 0.4 nm, as shown from Figure S3B, and the highness (thickness) is much smaller than the width, indicating the formation of a thin layer of the polymeric shell with a hollow core.

Variation in the size of the capsules was attempted by changing the amount of water phase to oil phase. To demonstrate this possibility, inverse miniemulsions were prepared by using the same amount of stabilizer with different water contents (iron salt was fixed at 2 wt % against water). When the amount of water phase increasing to 23 wt % to total weight, the size of resulted capsule is increased to ca. 60 to 80 nm with a broader particle size distribution as shown in Figure 1C. It can be anticipated that the particle size of the capsules can be rationally designed.

### 3.2. Synthesis and Characterization of PB Containing Polymeric Capsules

Potassium ferrocyanide was used as a co-stabilizer in the miniemulsion preparation step, which also played a role as a reactant in the formation of PB in the periphery of the crosslinked nanocapsule upon the addition of the FeCl_3_ ether solution to the emulsion system. Synthesis of PB containing polymer is schematically illustrated in Scheme 1. After miniemulsion crosslinking reaction, the amount of FeCl_3_ ether solution was injected into above medium. A visible color change from milky white to grayish blue was observed, indicating the occurrence of the coordination reaction between K_4_Fe(CN)_6_ and FeCl_3_. We notice that there is no obvious phase separation during the addition of FeCl_3_ ether solution within the ratio of Fe^3+^/Fe^2+^ lower than 1/5. Further increasing the ratio of Fe^3+^/Fe^2+^ leads to emulsion demulsification. It should be noted that in our experiment, FeCl_3_ was dissolved in ether to prepare a stock solution, since FeCl_3_ has a poor solubility in oil phase (toluene) of miniemulsion. However, ether is well soluble in toluene and not miscible with water. The nature of ether and FeCl_3_ forces the in situ coordination reaction between Fe^2+^ and Fe^3+^ to proceed at the oil–water interface of the miniemulsion. It is also worth to mention that when a FeCl_3_ ether solution was introduced into a fresh prepared miniemulsion without crosslinking reaction, a rapid oil/water phase separation was observed, suggesting that the effective crosslinking is necessary to protect the nanocapsule structure from damaged by coordination reaction.

**Figure 2:**
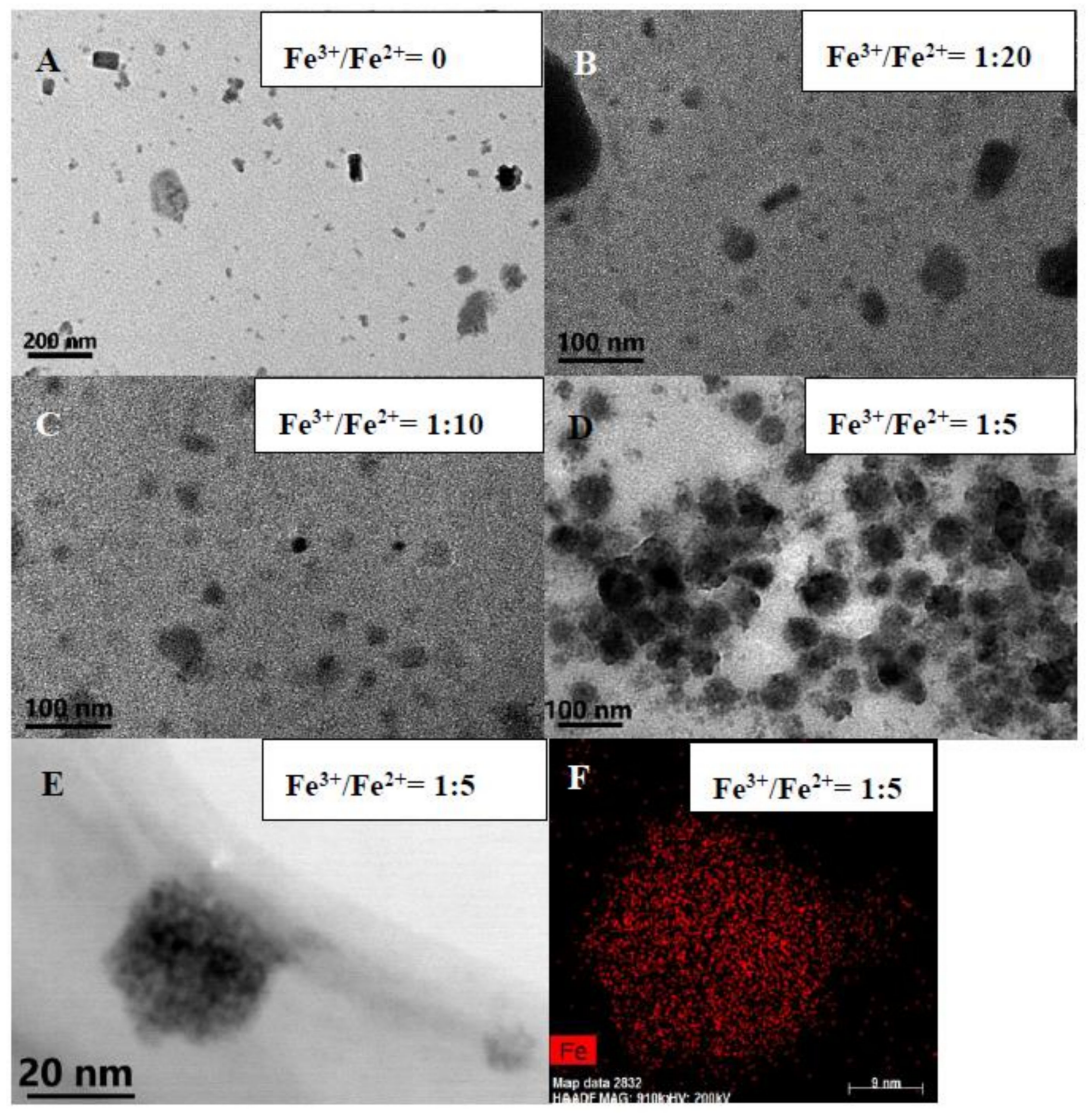
TEM images of samples prepared from PB containing capsules with different amount of added Fe^3+^. The amounts of added Fe^3+^ with respect to the Fe^2+^ are indicated in the images; Figure 2 (**A**–**D**) at lower magnification, (**E**) at higher magnification, and (**F**) EDX mapping of iron element.

We also investigated the coordination reaction with varied equivalents of Fe^3+^ with respect to Fe^2+^. In a set of four coordination experiments, 0, 1/5, 1/10 and 1/20 equivalents of Fe^3+^ in diethyl ether (0.0247 mol/L) with respect to Fe^2+^ (in water phase) of crosslinked miniemulsion were added to the emulsion system separately. For the experiment without added Fe^3+^ (Fe^3+^/Fe^2+^ = 0), the crosslinked nanocapsule only containing Fe^2+^ in water phase, which was diluted into methanol for TEM analysis without stain treatment (see Figure 2A). As shown, some irregular dark spots can be observed, which could come from K_4_Fe(CN)_6_ salt crystals in methanol rather than polymeric nanocapsules. Comparing with the same sample treated with negative stain as shown Figure 1A, which shows a uniform spherical nanostructure. As estimated from the image, the results suggest that nanoparticles without coordination reaction could not be revealed by TEM test due to the low contrast of polymer without staining. For the system, 1/20 equivalent of Fe^3+^ was added in relation to Fe^2+^ in crosslinked miniemulsion system. Spherical polymer particles appear, although the contrast is still low (see Figure 2B). Further increasing the molar ratio of Fe^3+^/Fe^2+^ to 1:10 and 1:5, the polymer spherical particles are becoming more and more clear as shown in Figure 2C,D respectively. The darker color of image is attributed to the PB coating, which have a higher electron density compared with the pure polymeric nanocapsule. Moreover, the resulted iron complex is evenly covered on the surface of the capsule, especially in Figure 2D. EDX mapping reveals that iron element (see Figure 2F) are uniformly distributed over the surface of the shell. The sample corresponding to Figure 2D was also characterized by DLS. The results revealed diameter of 229 nm with a broad size distribution 0.211 (see Figure S4, Supporting Information), which is larger than the crosslinked miniemulsion before coordination reaction. Coordination reaction may cause the particle join together as shown in Figure 2D. All these results indicate (1) a higher ratio of Fe^3+^/Fe^2+^ results a high density of PB and (2) the main site of the coordination reaction between two iron salts occurs at the surface of polymeric nanocapsules.

**Figure 3:**
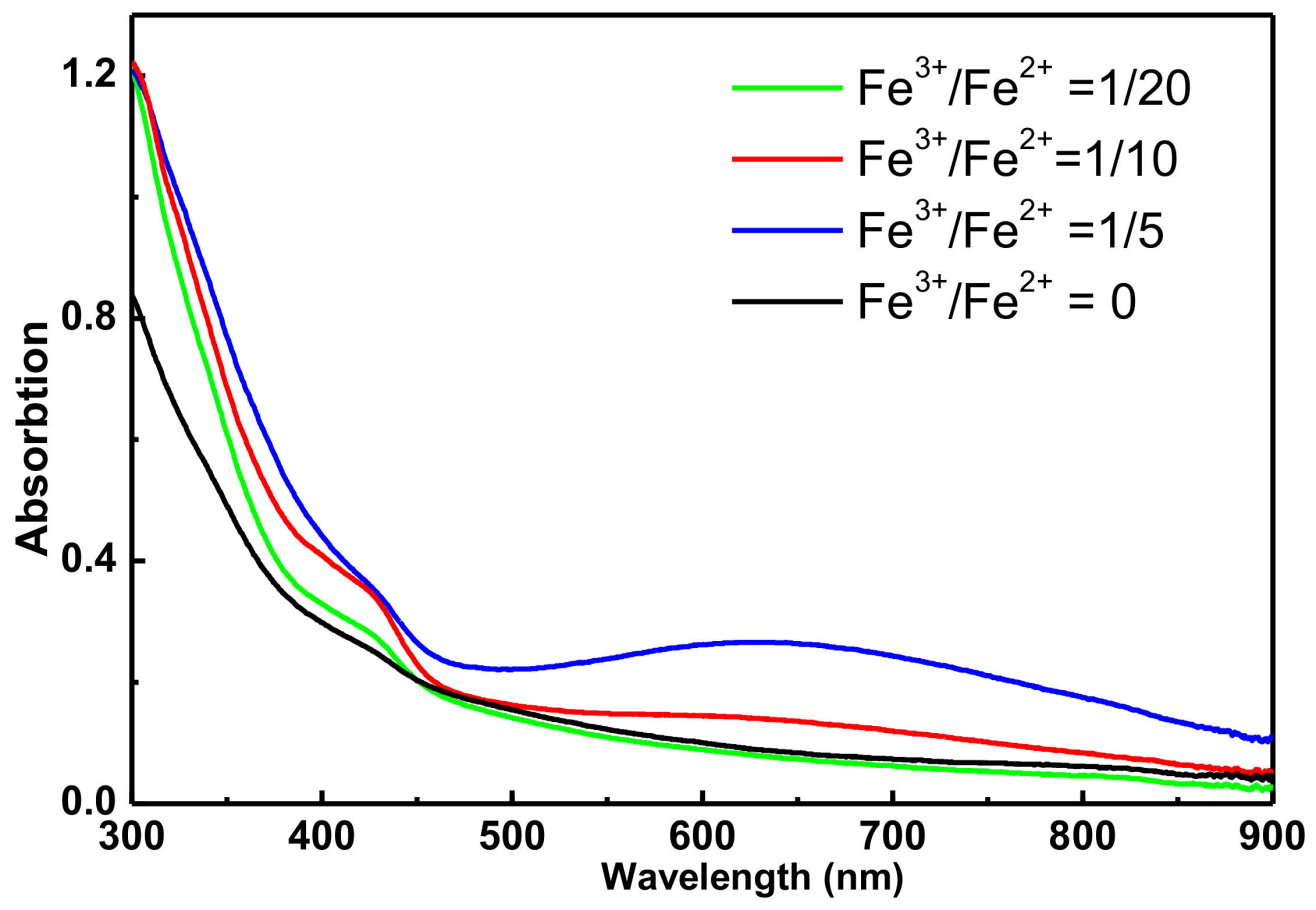
UV–Vis absorption spectra of the methanol solution of nanocapsule after addition various amount of Fe^3+^. The concentration of Fe^2+^ is 0.185 mg·mL^−1^.

**Figure 4:**
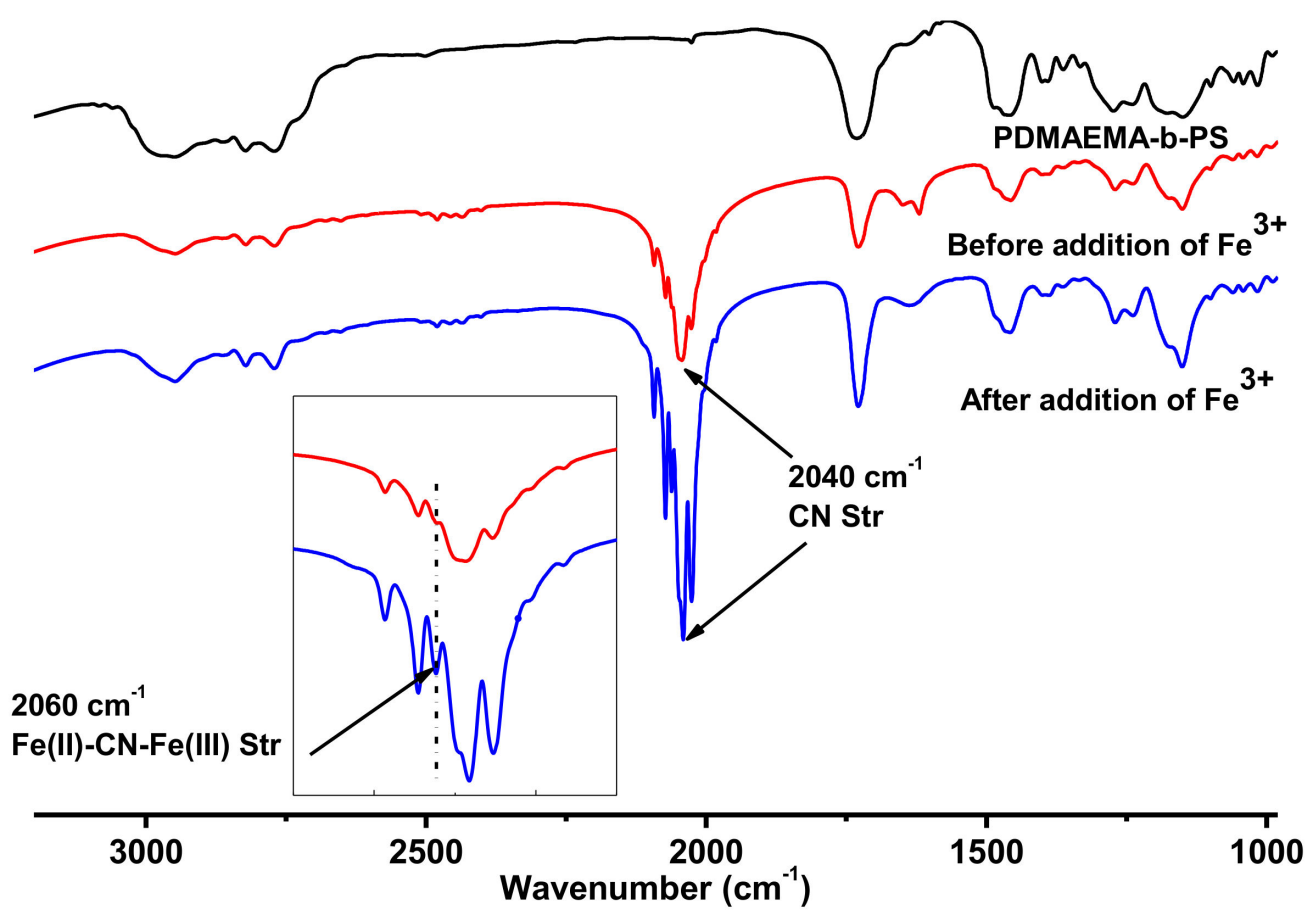
FT-IR spectra for PDMAEMA-b-PS, nanocapsules before addition of Fe^3+^ and after addition of Fe^3+^. The inserted box shows the enlargement of the curves (1900 to 2100 nm wavelength) of capsules before and after addition of Fe^3+^.

The samples were prepared by diluting 1 g of crosslinked miniemulsion with molar ratio of Fe^3+^/Fe^2+^ = 0, 1/5, 1/10 and 1/20 respectively into 10 g of methanol prior to UV–Vis spectroscopy testing. In them, the concentration of Fe^2+^ is 0.185 mg·mL^−^^1^. The results are shown in Figure 3. With the increasing of the amount of Fe^3+^ into crosslinked nanocapsule, a distinct absorbance at 600–800 nm appeared as shown in the Figure 3, ascribed to charge transfer of Fe(II)-CN-Fe(III) of the formed PB [21]. However, the absorbance signal is not high, it is due to the low concentration of PB (lower than 0.074 mg·mL^−1^). This phenomenon was also observed in Liang’s report [23]. Another possible reason which may affect the signal is that the system is heterogeneous due to particle stacking induced by coordination. In the FT-IR spectra (Figure 4), a strong absorption at 2040 cm^−1^ was observed both in curve of nanocapsules before addition of Fe^3+^ and in the curve of after addition of Fe^3+^, due to the CN stretching mode in K_4_Fe(CN)_6_. For the sample of nanocapsules after addition of Fe^3+^, there is still an excess of K_4_Fe(CN)_6_ salt dose not react. However, there is a new absorbance band at 2060 cm^−1^ was observed for nanocapsules after addition for Fe^3+^, due to the formation of a Fe(II)-CN-Fe(III) bond. 

**Figure 5:**
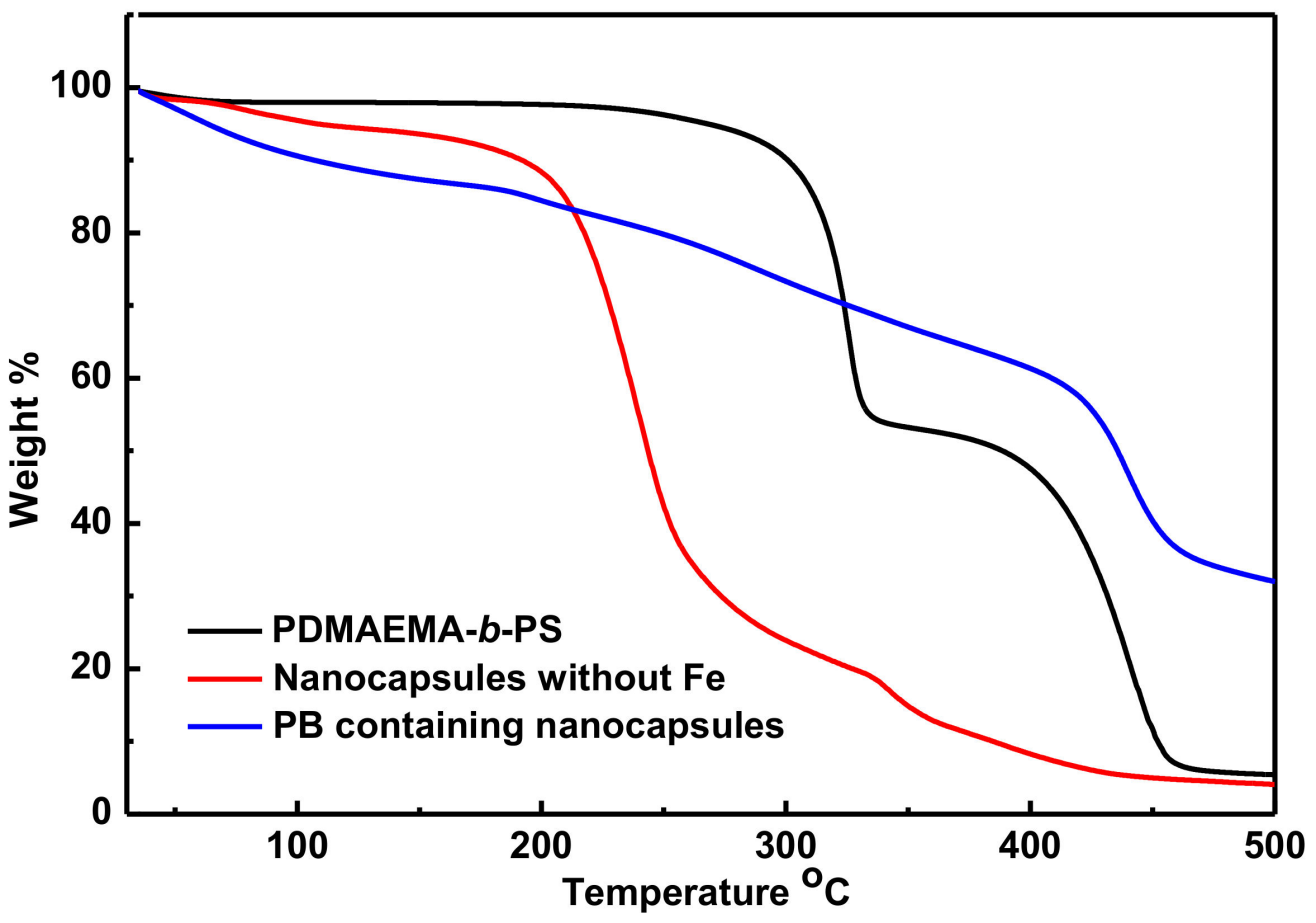
TGA traces of PDMAEMA-b-PS, nanocapsules without Fe and PB containing nanocapsules.

The thermostability of the PDMAEMA-*b*-PS, nanocapsules without Fe and PB containing capsules was evaluated using thermogravitic analysis (TGA) (see Figure 5). A polymeric nanocapsule were prepared without addition of iron salt in their preparation. Compared with PDMAEMA-*b*-PS, a lower thermal degradation temperature for nanocapsule without Fe was observed due to the easy loss of anion and ammonium groups of quanternized PDMAEMA (Hofmann elimination) [28]. However, the capsules containing PB shows that the capsules possessed improved thermal stability contrasting with its polymeric capsules without Fe salt. This phenomenon further illustrates that the PB formed on the polymer shell of the capsules, which prevents the polymer shell during thermal decomposition.

## 4. Conclusions

This work presents a robust method to achieve the synthesis of PB containing polymeric nanocapsules by two step experiments. In the first stage, a polymeric nanocapsule with uniform size was prepared successfully from PDMAEMA-*b*-PS stabilized inverse miniemulsion via BIEE quaternization crosslinking reaction. The size of nanocapsule could be adjusted by varying the ratio of oil phase to water phase in inverse miniemulsion. In the second stage, FeCl_3_ ether solution was introduced into the above crosslinked miniemulsion. With the reaction between K_4_Fe(CN)_6_ salt in its water core of emulsion and FeCl_3_ ether solution in the oil phase, the generation of PB containing polymeric nanocapsule was achieved. Due to the limited solubility of FeCl_3_ in the oil phase, forcing the coordination reaction between two iron salt occurred mainly in the oil/water interface (shell of nanocapsules) to form a PB containing polymeric nanostructure.

## Figures and Tables

**Table 1:** Characteristics of the PDMAEMA-CTA and PDMAEMA-b-PS block copolymers.

Polymer^a)^	*M*_theo._(g·mol^−1^)	*M*_n_(g·mol^−1^)^d)^	PDI^d)^
PDMAEMA_133_-CTA	21,102 ^b)^	25,000	1.20
PDMAEMA_133_-b-PS_28_	24,014 ^c)^	27,200	1.24

^a)^ The subscript number denotes the degree of polymerization (DP); ^b)^ Determined by comparison of the intensity of the end group protons resonating at δ = 7.4 to 8.1 to the intensity of the resonance from δ = 2.2 to 2.4 from PDMAEMA in the ^1^H-NMR spectra (Figure S1A, Supporting Information); ^c)^ Determined by comparison of the intensity of the protons resonating at δ = 3.8 to 4.2 to the intensity of the resonance from δ = 6.3 to 7.2 in Figure S1B; ^d)^ Molecular weight and polydispersities (PDI) of polymers were determined by SEC. The SEC system was calibrated with polystyrene standards.

## Supplementary Materials

The following are available online at http://www.mdpi.com/2073-4360/11/2/266/s1, Scheme S1: Synthetic scheme for the preparation of PDMAEMA-CTA and PDMAEMA-*b*-PS copolymer, Figure S1: ^1^H-NMR spectrum of poly(2-dimethylamino)ethyl methacrylate) PDMAEMA-CTA in CDCl_3_(A) and ^1^H-NMR spectrum of poly(2-dimethylamino)ethyl methacrylate)-block-polystyrene in CDCl_3_ (B). Figure S2. Diameter and polydispersity of cross-linked inverse miniemulsion in methanol. Inverse miniemulsion formulation; PDMAEMA-*b*-PS 0.065 g, toluene 6.5 g, distilled water 0.65 g, K_4_Fe(CN)_6_·3H_2_O 0.013 g, BIEE 0.1 g. For DLS samples preparation, the cross-linked miniemulsion (0.2 g) was diluted into 10 g of methanol directly for DLS measurements. Figure S3. Height image(A)of tapping mode AFM micrograph is 0.4 × 0.4 µm^2^, and Topography vs Distance image (B) of selected particles as red highlight in (A). Figure S4. Diameter and polydispersity of inverse miniemulsion after coordination reaction in methanol. PB coated nanocapsules prepared using crosslinked nanocapsules with Fe^3+^/Fe^2+^ = 1:5. For DLS samples preparation, the miniemulsion (0.2 g) was diluted into 10 g of methanol directly for DLS measurements.

## References

[1] Zhang Z., Zhang P., Wang Y., Zhang W. (2016). Recent advances in organic–inorganic well-defined hybrid polymers using controlled living radical polymerization techniques. Polym. Chem..

[2] Ha C.-S. (2017). Polymer Based Hybrid Nanocomposites; A Progress Toward Enhancing Interfacial Interaction and Tailoring Advanced Applications. Chem. Rec..

[3] Zhang S., Geryak R., Geldmeier J., Kim S., Tsukruk V.V. (2017). Synthesis, Assembly, and Applications of Hybrid Nanostructures for Biosensing. Chem. Rev..

[4] Shi J., Jiang Y., Wang X., Wu H., Yang D., Pan F., Su Y., Jiang Z. (2014). Design and synthesis of organic–inorganic hybrid capsules for biotechnological applications. Chem. Soc. Rev..

[5] Horecha M., Kaul E., Horechyy A., Stamm M. (2014). Polymer microcapsules loaded with Ag nanocatalyst as active microreactors. J. Mater. Chem. A.

[6] Gong M.-Q., Wu J.-L., Chen B., Zhuo R.-X., Cheng S.-X. (2015). Self-Assembled Polymer/Inorganic Hybrid Nanovesicles for Multiple Drug Delivery To Overcome Drug Resistance in Cancer Chemotherapy. Langmuir.

[7] Singh I., Landfester K., Chandra A., Munoz-Espi R. (2015). A new approach for crystallization of copper(ii) oxide hollow nanostructures with superior catalytic and magnetic response. Nanoscale.

[8] Cai W., Wang J., Chu C., Chen W., Wu C., Liu G. (2018). Metal–Organic Framework-Based Stimuli-Responsive Systems for Drug Delivery. Adv. Sci..

[9] Liu Y., Wang X. (2011). Recent advances in block copolymer-assisted synthesis of supramolecular inorganic/organic hybrid colloids. Polym. Chem..

[10] Teo G.H., Kuchel R.P., Zetterlund P.B., Thickett S.C. (2016). Polymer-inorganic hybrid nanoparticles of various morphologies via polymerization-induced self assembly and sol–gel chemistry. Polym. Chem..

[11] Liu Y., Wang X. (2012). Synthesis, characterization, micellization and metal coordination polymerization of pentacyanoferrate-coordinated block copolymers for monodispersed soluble Prussian blue nanospheres. Polym. Chem..

[12] Wang X., Jiang Z., Shi J., Liang Y., Zhang C., Wu H. (2012). Metal–Organic Coordination-Enabled Layer-by-Layer Self-Assembly to Prepare Hybrid Microcapsules for Efficient Enzyme Immobilization. ACS Appl. Mater. Interfaces.

[13] Benedetti C., Cazzolaro A., Carraro M., Graf R., Landfester K., Gross S., Muñoz-Espí R. (2016). Dual Role of Zirconium Oxoclusters in Hybrid Nanoparticles: Cross-Linkers and Catalytic Sites. ACS Appl. Mater. Interfaces.

[14] Srivastava S., Schaefer J.L., Yang Z., Tu Z., Archer L.A. (2013). 25th Anniversary Article: Polymer–Particle Composites: Phase Stability and Applications in Electrochemical Energy Storage. Adv. Mater..

[15] Bhatt P., Meena S.S., Mukadam M.D., Mandal B.P., Chauhan A.K., Yusuf S.M. (2018). Synthesis of CoFe Prussian blue analogue/poly vinylidene fluoride nanocomposite material with improved thermal stability and ferroelectric properties. New J. Chem..

[16] Ye S., Liu Y., Chen S., Liang S., McHale R., Ghasdian N., Lu Y., Wang X. (2011). Photoluminescent properties of Prussian Blue (PB) nanoshells and polypyrrole (PPy)/PB core/shell nanoparticles prepared via miniemulsion (periphery) polymerization. Chem. Commun..

[17] Uemura T., Ohba M., Kitagawa S. (2004). Size and Surface Effects of Prussian Blue Nanoparticles Protected by Organic Polymers. Inorg. Chem..

[18] McHale R., Ghasdian N., Liu Y., Wang H., Miao Y., Wang X. (2010). Synthesis of Prussian Blue Coordination Polymer Nanocubes via Confinement of the Polymerization Field Using Miniemulsion Periphery Polymerization (MEPP). Macromol. Rapid Commun..

[19] Liang G., Ni H., Bao S., Zhu F., Gao H., Wu Q. (2014). Synthesis and Characterization of Nanowire Coils of Organometallic Coordination Polymers for Controlled Cargo Release. J. Phys. Chem. B.

[20] Uemura T., Kitagawa S. (2003). Prussian Blue Nanoparticles Protected by Poly(vinylpyrrolidone). J. Am. Chem. Soc..

[21] Liang G., Xu J., Wang X. (2009). Synthesis and Characterization of Organometallic Coordination Polymer Nanoshells of Prussian Blue Using Miniemulsion Periphery Polymerization (MEPP). J. Am. Chem. Soc..

[22] McHale R., Ghasdian N., Liu Y., Ward M.B., Hondow N.S., Wang H., Miao Y., Brydson R., Wang X. (2010). Prussian blue coordination polymer nanobox synthesis using miniemulsion periphery polymerization (MEPP). Chem. Commun..

[23] Liang G., Li X., Fei B., Wang X., Zhu F. (2015). Tiny nanoparticles of organometallic polymers through the direct disassembly-assisted synthesis strategy for hydrogen peroxide sensing. Polym. Chem..

[24] Wu L., Pang T., Guan Y.-B. (2016). Miniemulsion cross-linking: A convenient route to hollow polymeric nanocapsule with a liquid core. Chin. J. Polym. Sci..

[25] Wu L., Li Y., Pang T., Guan Y.-B. (2017). One-pot synthesis of PDMAEMA nanocapsules for controlled release of hydrophobic cargo. J. Macromol. Sci. Part A.

[26] Liang G., Ni H., Bao S., Zhu F., Gao H., Wu Q., Tang B.Z. (2014). Amphiphilic Nanocapsules Entangled with Organometallic Coordination Polymers for Controlled Cargo Release. Langmuir.

[27] Jiang G., Wang Y., Zhang R., Wang R., Wang X., Zhang M., Sun X., Bao S., Wang T., Wang S. (2012). Preparation of Redox-Sensitive Shell Cross-Linked Nanoparticles for Controlled Release of Bioactive Agents. ACS Macro Lett..

[28] Roy D., Knapp J.S., Guthrie J.T., Perrier S. (2008). Antibacterial Cellulose Fiber via RAFT Surface Graft Polymerization. Biomacromolecules.

