# Supplementary material for: Preparation of Prussian Blue Containing Polymeric Nanocapsule via Interfacial Confined Coordination in Crosslinked Inverse Miniemulsion
Source: Polymers (Basel). 2019 Feb 5;11(2):266. doi: 10.3390/polym11020266 (PMC6419073; doi:10.3390/polym11020266)
Supplement: Supplementary file 1 [file polymers-11-00266-s001.pdf]

Supporting Information

Preparation of Prussian blue containing polymeric nanocapsule via interfacial confined coordination in crosslinked inverse miniemulsion

Lin Wu[a]*, Tao Pang[a] , Yebin Guan[a], Yiguo Li[bc]*

[a]Anhui Key laboratory of functional coordination compounds, School of chemistry and chemical engineering, Anqing Normal University, Anqing, Anhui, 246011, China
[b]Ningbo key laboratory of specialty polymers, Faulty of materials science and chemical engineering, Ningbo University, Ningbo, 315211, China
[c]Anhui Collaborative Innovation Centre for Petrochemical New Materials, School of Chemistry and Chemical Engineering, Anqing Normal University, Anqing, Anhui, 246011, China

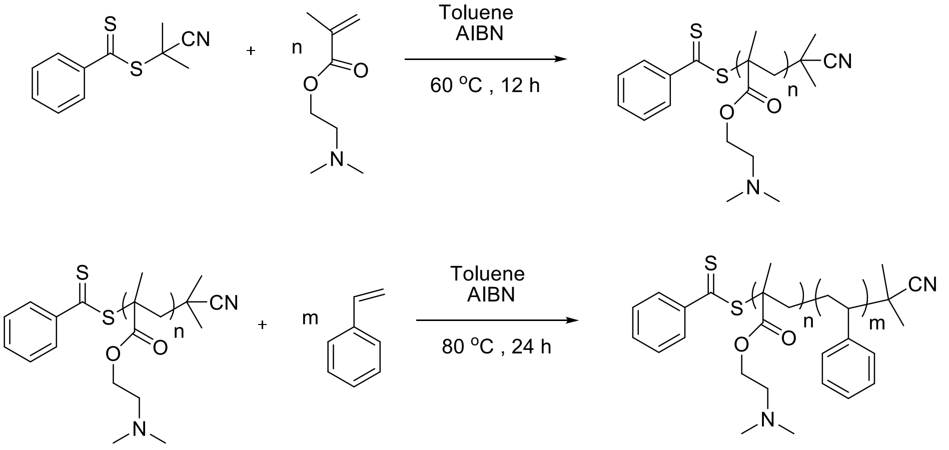

Scheme S1. Synthetic scheme for the preparation of PDMAEMA-CTA and PDMAEMA-*b*-PS copolymer.

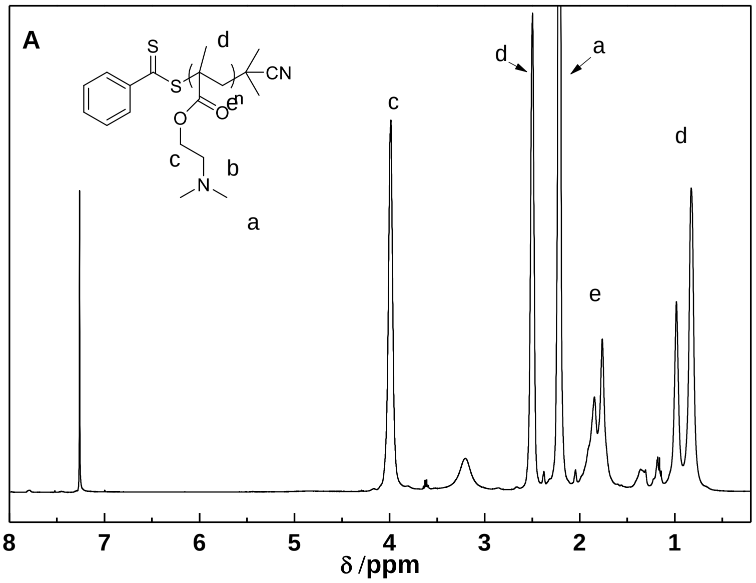

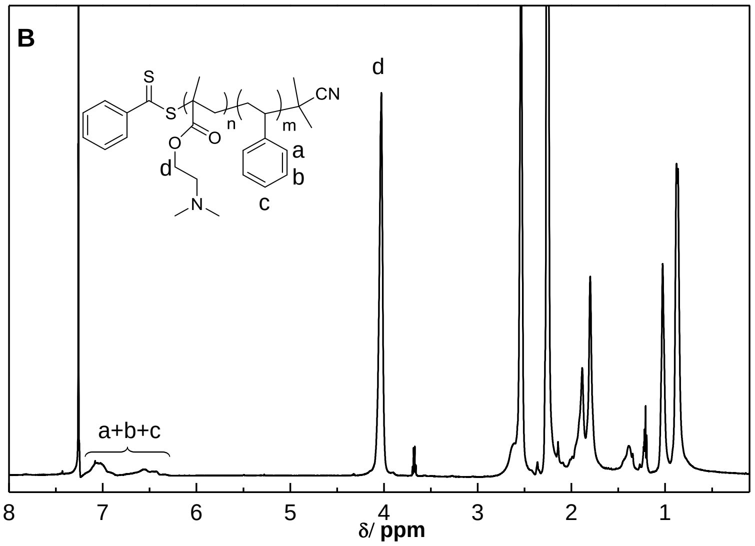

Figure S1. ^{1}H-NMR spectrum of poly((2-dimethylamino)ethyl methacrylate) PDMAEMA-CTA in $CDCl_3$(A) and ^{1}H-NMR spectrum of poly(2-dimethylamino)ethyl methacrylate)-block-polystyrene in $CDCl_3$ (B) .

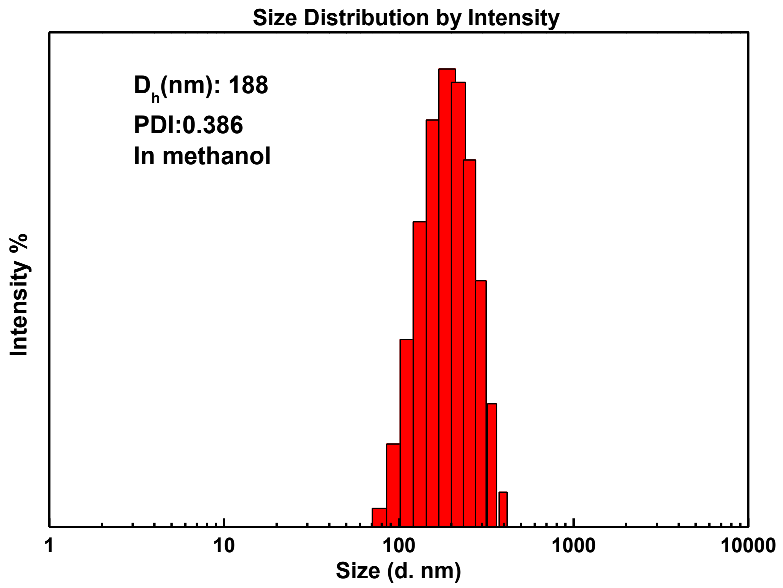

Figure S2. Diameter and polydispersity of cross-linked inverse miniemulsion in methanol. Inverse miniemulsion formulation; PDMAEMA-*b*-PS 0.065 g, toluene 6.5 g, distilled water 0.65 g, $K_4Fe(CN)_6 \cdot 3H_2O$ 0.013 g, BIEE 0.1 g. For DLS samples preparation, the cross-linked miniemulsion (0.2 g) was diluted into 10 g of methanol directly for DLS measurements.

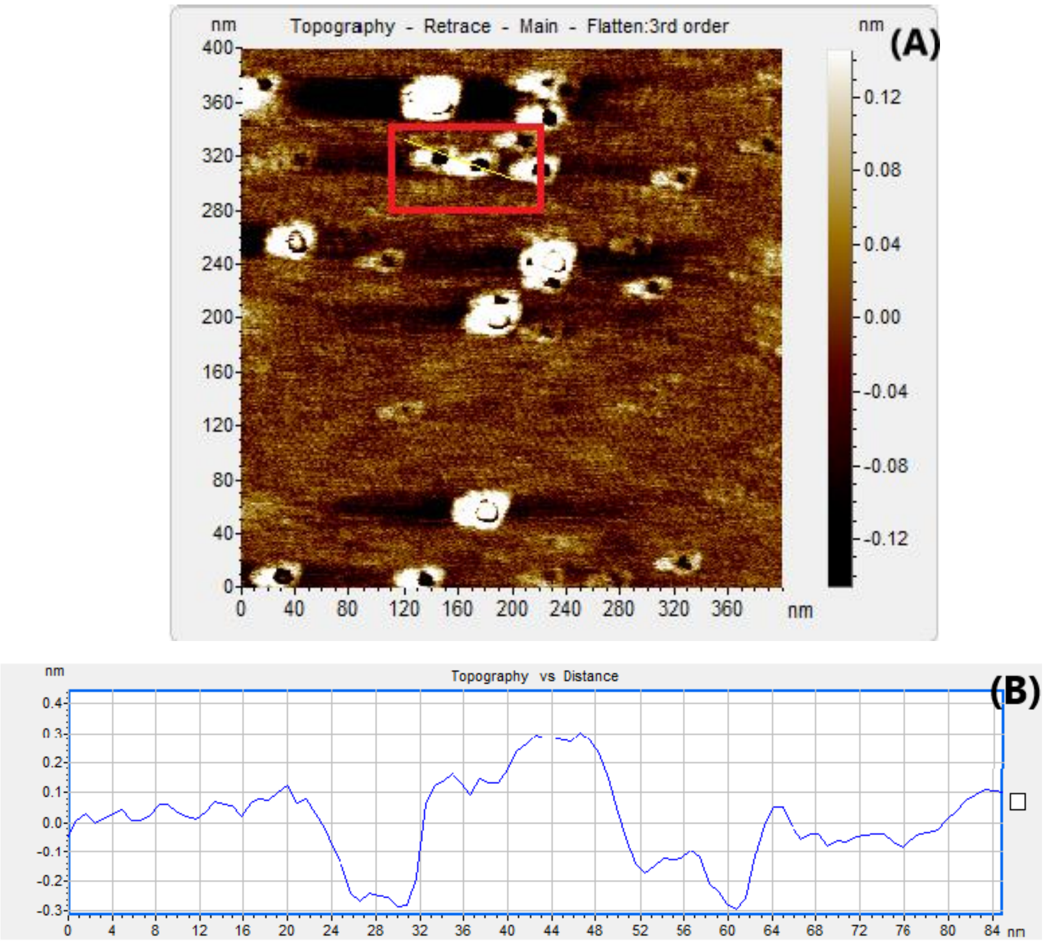

Figure S3. Height image(A)of tapping mode AFM micrograph is 0.4 × 0.4 μm^2, and Topography vs Distance image (B) of selected particles as red highlight in (A) .

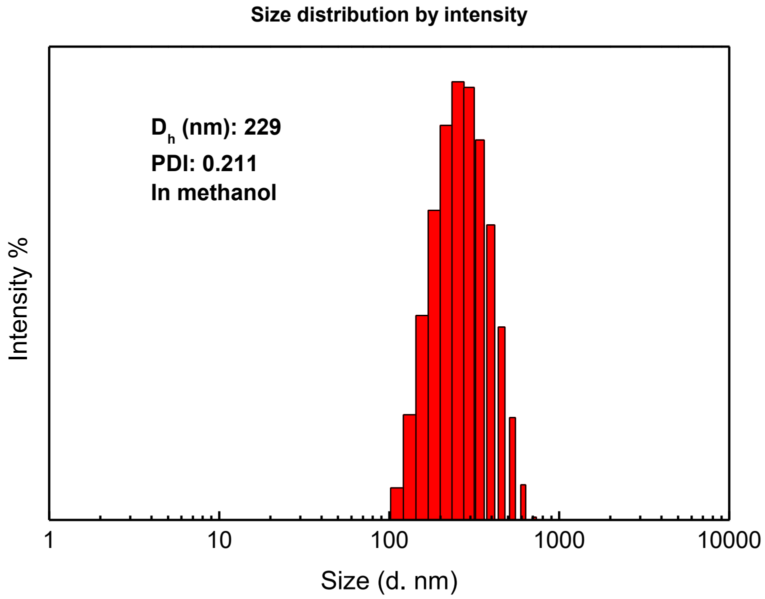

Figure S4. Diameter and polydispersity of inverse miniemulsion after coordination reaction in methanol. PB coated nanocapsules prepared using crosslinked nanocapsules with Fe^{3+}/Fe^{2+} = 1:5. For DLS samples preparation, the miniemulsion (0.2 g) was diluted into 10 g of methanol directly for DLS measurements.
